# Attenuation of neuroinflammation reverses Adriamycin-induced cognitive impairments

**DOI:** 10.1186/s40478-019-0838-8

**Published:** 2019-11-21

**Authors:** Barrett D. Allen, Lauren A. Apodaca, Amber R. Syage, Mineh Markarian, Al Anoud D. Baddour, Harutyun Minasyan, Leila Alikhani, Celine Lu, Brian L. West, Erich Giedzinski, Janet E. Baulch, Munjal M. Acharya

**Affiliations:** 1 Department of Radiation Oncology, University of California, Medical Sciences I, Room B-149, Irvine, CA 92697-2695 USA; 2 Plexxikon Inc., Berkeley, CA USA

**Keywords:** Chemotherapy, Adriamycin, Doxorubicin, Chemobrain, Neuroinflammation, Cognitive dysfunction, Colony stimulating factor receptor 1, Microglia, Extracellular vesicles, Induced pluripotent stem cells

## Abstract

Numerous clinical studies have established the debilitating neurocognitive side effects of chemotherapy in the treatment of breast cancer, often referred as chemobrain. We hypothesize that cognitive impairments are associated with elevated microglial inflammation in the brain. Thus, either elimination of microglia or restoration of microglial function could ameliorate cognitive dysfunction. Using a rodent model of chronic Adriamycin (ADR) treatment, a commonly used breast cancer chemotherapy, we evaluated two strategies to ameliorate chemobrain: *1)* microglia depletion using the colony stimulating factor-1 receptor (CSF1R) inhibitor PLX5622 and *2)* human induced pluripotent stem cell-derived microglia (iMG)-derived extracellular vesicle (EV) treatment. In strategy 1 mice received ADR once weekly for 4 weeks and were then administered CSF1R inhibitor (PLX5622) starting 72 h post-ADR treatment. ADR-treated animals given a normal diet exhibited significant behavioral deficits and increased microglial activation 4–6 weeks later. PLX5622-treated mice exhibited no ADR-related cognitive deficits and near complete depletion of IBA-1 and CD68^+^ microglia in the brain. Cytokine and RNA sequencing analysis for inflammation pathways validated these findings. In strategy 2, 1 week after the last ADR treatment, mice received retro-orbital vein injections of iMG-EV (once weekly for 4 weeks) and 1 week later, mice underwent behavior testing. ADR-treated mice receiving EV showed nearly complete restoration of cognitive function and significant reductions in microglial activation as compared to untreated ADR mice. Our data demonstrate that ADR treatment elevates CNS inflammation that is linked to cognitive dysfunction and that attenuation of neuroinflammation reverses the adverse neurocognitive effects of chemotherapy.

## Introduction

The clinical benefits of chemotherapy are achieved through acute cytotoxicity, however this toxicity also translates into chronic adverse neurocognitive outcomes, often referred to as *chemobrain* [7]. This is particularly prevalent in breast cancer survivors, the most common form of invasive cancer in women. Currently, there are over 3.1 million breast cancer survivors in the U.S. and about 268,000 new cases will be diagnosed each year [11]. Many of these breast cancer survivors (17 to 75%) experience subtle to severe emotional, behavioral and cognitive decrements that affect their ability to concentrate, plan, multitask and remember [42]. For these reasons cognitive status is now, after survival, considered the most important clinical criterion for evaluating therapeutic outcome. The conspicuous absence of mitigation strategies for reducing the progressive neurocognitive side effects represents a critical unmet medical need and, breast cancer survivors represent a significant patient base whose quality of life would be improved by therapeutic interventions.

Pre-clinical chemobrain models have established the neurocognitive and neurobiological consequences of commonly used chemotherapeutic agents for breast cancer therapy [reviewed in [40]]. We have shown that chronic cyclophosphamide (CYP) or doxorubicin (Adriamycin, ADR) monotherapy severely impairs hippocampal- and frontal cortex-dependent cognitive function in rodents [5, 22] that were linked with decline in neurogenesis, mature neuron structure damage and persistent inflammation (i.e. microglial activation). Acute ADR exposure was associated with reduced hippocampal LTP, elevated lipid peroxidation and apoptosis [9]. Despite the very low penetrance of CYP or ADR across the blood brain barrier (BBB), acute CYP or ADR treatments negatively impacted hippocampal cell proliferation and increased cell death demonstrating the extreme sensitivity of the CNS to chemotherapy [32, 51]. In rodents, combined ADR and CYP treatment impaired contextual fear conditioning memory and passive avoidance tasks and, elevated oxidative stress and inflammation [13, 35]. These studies suggest that exposure of the brain parenchyma to even low levels of drug may be sufficient to disrupt sensitive, rapidly dividing cells in neurogenic regions and elevate neuroinflammation long after cessation of chemotherapy. Pre-clinical and clinical reports also suggest that treatment with ADR acutely elevates plasma TNFα that may perturb the integrity of the BBB and exacerbate inflammatory cascades in the CNS leading to brain injury [48].

Pharmacological and non-pharmacological interventions to alleviate chemobrain have shown only marginal benefits [reviewed in [40]]. These include neuropsychological or cognitive behavioral therapy, physical exercise and treatment with drugs targeting neurotransmitter systems, albeit the pharmacological approach was also associated with significant side effects. Based on our past studies using rodent models of cancer therapy (e.g. irradiation and/or chemotherapy)-related cognitive impairments we hypothesize that persistent neuroinflammation is one of the major drivers of brain injury and cognitive dysfunction [3–6, 17]. Consequently, using a cranial radiation-induced brain injury model, we showed that dietary treatment with a colony stimulating factor-1 receptor (CSF1R) inhibitor (PLX5622) [4], stem cells [3], or stem cell-derived extracellular vesicle (EV) [17] transplantation ameliorated behavioral impairments in the irradiated animals. A number of CSF1R inhibitors are currently under evaluation in the clinic for cancer therapy [21]. Moreover, stem cell-derived EVs protected from radiation-induced CNS inflammation – a parallel neuropathology reported in chemobrain models [17]. In this report, we now show the beneficial neurocognitive and anti-inflammatory effects of two distinct strategies including a dietary treatment with the CSF-1R inhibitor PLX5622 and intravenous injections of EV isolated from human induced pluripotent stem cell (iPSC)-derived microglia (iMG) in a mouse model of ADR-induced cognitive impairments.

## Materials and methods

Details on materials, experimental methods, behavior and immunostaining protocols are provided in the Additional file 1 section.

### Animals and treatments

**Fig. 1:**
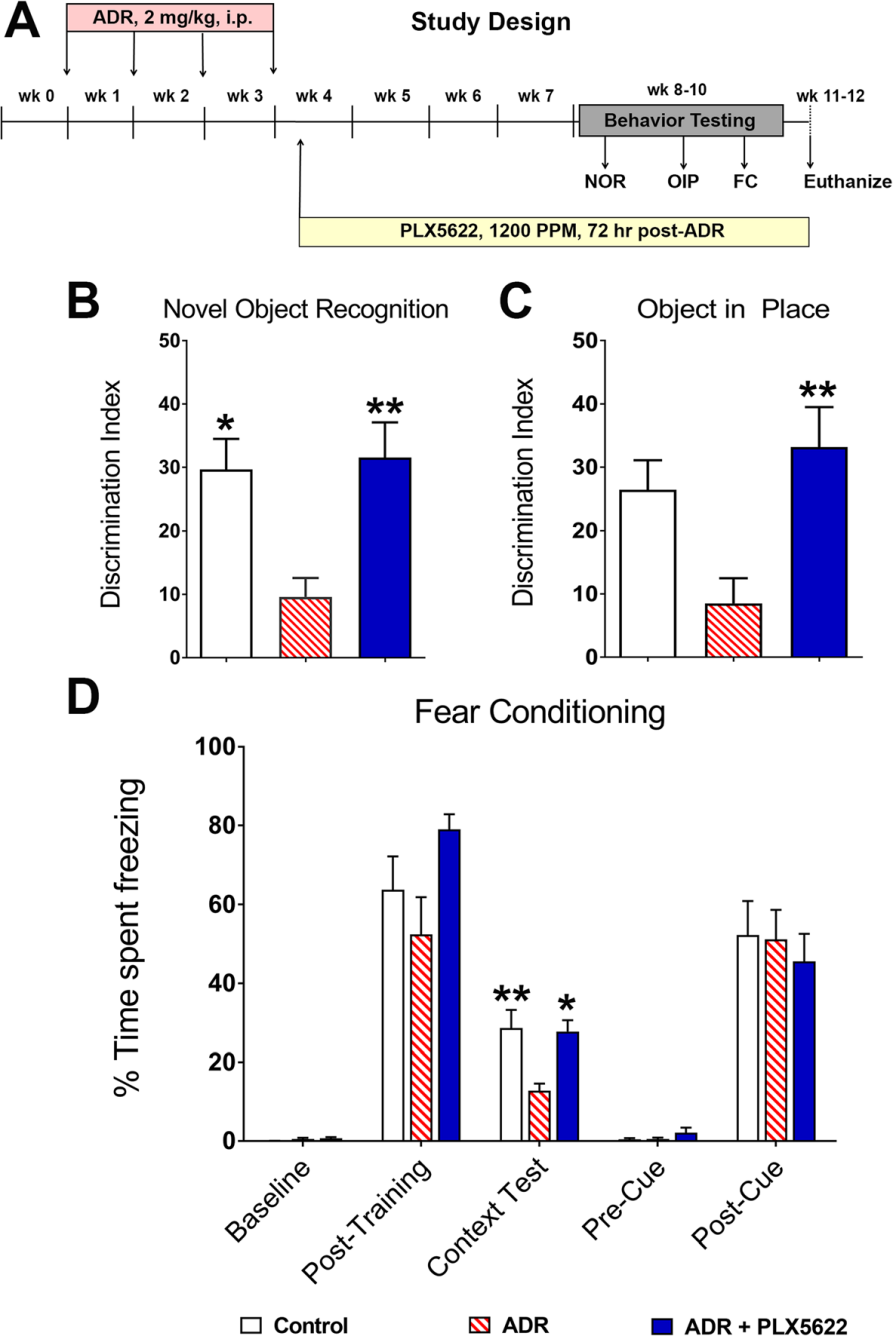
Treatment with CSF1R inhibitor reverses Adriamycin-induced cognitive impairments. **a**, Schematic presentation of the study design: Six-month old wild type (C57BL/6 J) male mice were injected with vehicle or Adriamycin (ADR, 2 mg/kg, i.p.), once weekly for 4 weeks. 72 h after the last ADR injection, mice began treatment with the CSF1R inhibitor PLX5622 in rodent chow and continued on diet till the end of the study. ADR-treated animals that received control chow served as vehicle group. One month after initiation of PLX5622 treatment, mice were administered spatial and episodic memory retention testing using the novel object recognition (NOR) and object in place (OIP) tasks followed by fear conditioning (FC) task. After completion of cognitive testing brains were collected for immunohistochemistry, RNA sequencing and cytokine analyses. **b, c** The tendency to explore novel spatial locations or objects was derived from the Discrimination Index, calculated as ([Novel object exploration time/Total exploration time] – [Familiar object exploration time/Total exploration time]) × 100. Chronic treatment with ADR significantly impaired cognitive function. Preference towards the novel object (NOR task, *, *P’s* < 0.01 compared to ADR group, **b, c**) was significantly reduced in the ADR-treated group receiving control diet. In contrast, ADR-treated mice receiving the PLX5622 diet showed significant improvements on the performance on both NOR and OIP tasks (**, *P’s* < 0.006 compared to ADR group, **b**, **c**). **d** Treatment with CSF1R inhibitor improves cognitive function on the hippocampal-dependent contextual fear-conditioning task. The baseline freezing levels were comparable among groups, and all groups (Control, ADR and ADR + PLX5622) showed elevated freezing behavior following a series of 5 tone-shock pairings (post-training). 24 h after fear conditioning training, the context test was administered where the ADR-treated mice receiving control diet showed significantly decreased freezing compared to Controls (**, *P* < 0.006). ADR-treated mice receiving PLX5622 diet (ADR + PLX5622) showed a significant elevation in freezing behavior compared to ADR treated mice receiving the control diet (*, *P* < 0 .01), and the level of freezing was indistinguishable from the control group. After the initial training phase (48 h), the context (spatial environment) and the odor was changed that resulted in a considerable reduction in freezing behavior (Pre-Cue bars) that was restored when tone was played (Post-Cue test bars), indicating intact amygdala function in all groups. Data are presented as mean ± SEM (*N* = 10 mice per group). *P* values were derived from ANOVA and Bonferroni’s post hoc test

All animal procedures are approved by the Institutional Animal Care and Use Committee and, according to the federal (NIH) guidelines. Six-month old male wild type mice (C57BL/6 J, Jackson) received ADR (doxorubicin hydrochloride, Sigma) dissolved in saline (2 mg/kg, once weekly, i.p.) for 4 weeks as shown in the study design (Fig. 1a). For the CSF1R inhibition study, mice were divided into three experimental groups (*N* = 10–12 mice per group): saline treated control mice receiving control chow (Control), ADR-treated mice receiving control chow (ADR) and, ADR-treated mice receiving PLX5622 chow (ADR + PLX5622). 72 h after the last ADR injection mice were provided the control or CSF1R inhibitor. PLX5622 chow, was provided by Plexxikon (Berkeley, CA) and formulated in AIN-76A standard chow by Research Diets (New Brunswick, NJ) at a dose of 1200 PPM. Control mice received AIN-76A chow without PLX5622. The rationale for using male mice is based on our past chemobrain studies using Adriamycin and cyclophosphamide showing the detrimental neurocognitive and neurodegenerative effects of these drugs on CNS function [22]. We conducted this proof-of-concept countermeasure study using male mice given our established chemobrain model and cognitive testing protocols, and to avoid potential hormonal influences on cognitive function. All mice were maintained on their respective PLX5622 or control diet throughout the duration of the study.

EV were isolated from human iPSC-derived microglia. Human microglia (iMG) were differentiated by a simplified method from human iPSC-derived mesodermal, hematopoietic stem cells as described [1, 41]. RNA sequencing, phagocytosis, and transplantation studies validated the functional microglial characteristics of these cells [1, 41]. Briefly, large batches of conditioned medium were collected by Research and Development Laboratory, Cellular Dynamics, Inc. (Madison, WI) during the maturation phase (days 28 to 35) of the differentiated iMG culture and refrigerated conditioned media was shipped to UCI for the isolation of EV. EV isolation was carried out using the ultracentrifugation protocol as described in detail [17, 49]. EV quantity and size were determined using ZetaView PMX110 particle analyzer (Meerbusch, Germany). The iMG conditioned media yielded a total of 7.07 × 10^11^ EV per ml with the mean diameter of 65 nm. The purified EV were stored in sterile phosphate buffered saline (PBS, 100 mM, pH 7.4, Gibco) at 4 °C. Animals were divided into three groups (*N* = 8 mice per group): Controls receiving PBS (Controls), ADR-treated receiving PBS (ADR) and ADR receiving iMG-EV injection via retro-orbital sinus route of administration once weekly for 4 weeks (1.36 × 10^7^ EV per 50 μL per injection; ADR + iMG-EV). We did not observe significant effects of any of these treatments on animal body weights (Additional file 1: Figure S1).

### Cognitive testing

To determine the effect of CSF1R inhibition on cognitive function after chronic chemotherapy, mice were administered behavioral testing 4 weeks after the initiation of PLX5622 treatment. Testing spanned over 3 weeks including the spontaneous exploration tasks Novel Object Recognition (NOR) and Object in Place (OiP), followed by the contextual and cued fear conditioning (FC) task. The NOR task evaluates episodic recognition memory through measuring the preference of mice to investigate novel object environmental changes, whereas the OiP task evaluates associative recognition memory [15, 16]. The discrimination index was then calculated for each mouse from these values: [(Novel/Total exploration time) – (Familiar/Total exploration time)] × 100. A positive index indicates that animals spent more time exploring novelty. A negative score indicates that animals exhibited little or no preference for novelty. After completion of spontaneous exploration tasks, the FC task was administered in three sequential phases over 3 days including a training phase, a context test and a cue test as described previously [5, 22].

For the iMG-EV treatment study, cognitive function, including NOR and fear extinction (FE) memory testing, were carried out 1 week after the last EV injection (5 weeks after the last ADR treatment). NOR testing was carried out as described above. To determine if chronic chemotherapy or EV treatment affects hippocampal-dependent fear conditioning learning and memory consolidation, we performed a series of FE assays modified to be reliant on hippocampal function (see Additional file 1 for details). On first day of conditioning, animals were presented with three pairing of auditory stimulus co-terminating with a mild foot shock. On the following 2 days (extinction training), animals were presented with 20 tones in the same contextual environment (odor and cues). On the final day of fear testing, animals were presented with only three tones in the same context. Freezing behavior was recorded using ceiling-mounted camera in the test chamber and scored by an automated measurement program (FreezeFrame, Coulbourn Instruments). The percentage of time each mouse spent freezing during the tone was then calculated for each phase of the fear response testing.

### Immunohistochemistry, confocal microscopy and volumetric quantification

After completion of behavioral testing, mice were deeply anesthetized using isoflurane and euthanized via intercardiac perfusion using saline with heparin (10 U/ml, Sigma) followed by 4% paraformaldehyde in PBS (ACROS Organics, NJ). Coronal brain sections (30 μm thick, 3–4 sections per brain) from each of 4–6 animals per experimental group were selected for the immunofluorescence analysis of microglia (IBA-1 and CD68) as described [3, 4]. Confocal z stacks were collected for the quantification of IBA-1^+^ and CD68^+^ cells using 3D algorithm-based volumetric analyses (AutoQuantX3, MediaCybernetics and, Imaris, v9.2, Bit Plane Inc., Switzerland) as described [2, 4]. Data are expressed as mean immunoreactivity (percentage) relative to the vehicle-treated controls.

### Cytokine and gene expression analyses

Freshly dissected hippocampi from each brain (*N* = 3–5 per group) were homogenized, washed and supernatants were shipped to Quansys Biosciences (Logan, UT) for the multiplex cytokines analysis using Q-Plex 14 cytokine array kit. Positive readouts were reported and plotted as the mean ± SEM. For the gene expression analysis, total mRNA was extracted and, microglial function and pro-inflammatory genes were analyzed using the NanoString mouse immunology panel (NanoString Technologies). Gene expression values were presented as percentage of vehicle-treated control group.

### Statistical analysis

Statistical analyses were carried out using GraphPad Prism (v6). One-way ANOVA were used to assess significance between the groups. When overall group effects were found to be statistically significant, a Bonferroni’s multiple comparisons test was used to compare the ADR with individual experimental groups. For analysis of fear conditioning and fear extinction data, repeated measures two-way ANOVA were performed. Wilcoxon matched-pairs signed rank test was used to compare exploration of same animals with familiar versus novel objects or places and, freezing behavior during the day of extinction training versus test phases. All analyses considered a value of *P* ≤ 0.05 to be statistically significant.

## Results

### CSF1R inhibition mitigates ADR treatment-induced behavioral impairments

One month after initiation of PLX5622 treatment (Fig. 1a), mice were habituated and tested on the NOR task (Fig. 1b). For the test phase, a significant overall group difference was found between the treatment cohorts for the discrimination index (F_(2, 27)_ = 7.04, *P* = 0.004). After a five-minute retention interval in the home cage, ADR animals spent a significantly lower proportion of time exploring the novel object compared to Controls (*P* = 0.01) and ADR + PLX5622 (*P* = 0.007) groups (Fig. 1b). Conversely, ADR + PLX5622 treated animals did not differ from Control animals. We did not include a Control + PLX5622 group as past reports from our laboratory and others show that short- or long-term treatment with PLX5622 did not affect cognitive function in intact, control animals [4, 24, 43]. After NOR testing, animals were habituated and tested in the OiP arena. During the OiP test phase, a significant group difference was found between the treatment cohorts for the discrimination index (F_(2, 27)_ = 6.36, *P* = 0.006). Control and ADR + PLX5622 cohorts showed comparable preference for the objects placed at novel locations (Fig. 1c) whereas ADR-treated animals receiving control diet showed significantly less preference to novel locations compared to ADR + PLX5622 animals (*P* = 0.006). For each of the above open arena tasks, the overall tendency of ADR-treated cohorts was to explore less during the NOR and OIP phases (Additional file 1: Figure S2). Our past data evaluating CYP-induced cognitive impairments showed similar reductions in the total exploration times compared to control animals [5]. Though, reduced time exploring the objects was less likely to impact discrimination between the novel and familiar object. Additionally, Wilcoxon matched-pairs signed rank tests comparing familiar and novel exploration times revealed significant effects for the Control (*P* = 0.002) and ADR + PLX5622 (*P* = 0.002) for both NOR and OIP tests whereas differences for the ADR group were not statistically significant.

Our past data showed impaired contextual fear memory after chronic chemotherapy [5, 22]. Thus, to ascertain if CSF1R inhibition exert beneficial effects on the fear memory, animals were administered fear conditioning task. Each phase of the FC task (training, cue and context tests) were administered over 3 days. Repeated measures ANOVA showed a significant overall group × phase interaction effect for the percentage of time spent freezing during the FC task (Fig. 1d; F_(4, 108)_ = 103.4, *P* = 0.0001). Repeated measures two-way ANOVA for each phase revealed significant differences between ADR and ADR + PLX5622 groups in the post-training (*P* = 0.001) and context (*P* = 0.01) phases. Groups did not differ significantly in freezing behavior across baseline, pre-cue, and post-cue phases, indicating a selective deficit in the hippocampal-dependent contextual memory phase of the task. The extent of freezing observed during the context phase is similar to that reported by our groups and others in the field [5, 22, 44, 50]. During the context test phase, post hoc tests confirmed that ADR animals spent significantly decreased percentages of time freezing compared with Control (*P* = 0.006) and ADR + PLX5622 (*P* = 0.01) groups, whereas Control and ADR + PLX5622 groups did not differ. Moreover, all groups showed significant increases in freezing behavior after the tone-shock pairings (post-training phase) indicating that ADR treatment did not impair sensory function. These data corroborate our past findings that exposure to chemotherapy significantly impairs learning and memory function [5, 22].

### CSF1R inhibition reduced microglial activation in the ADR-treated brains

**Fig. 2:**
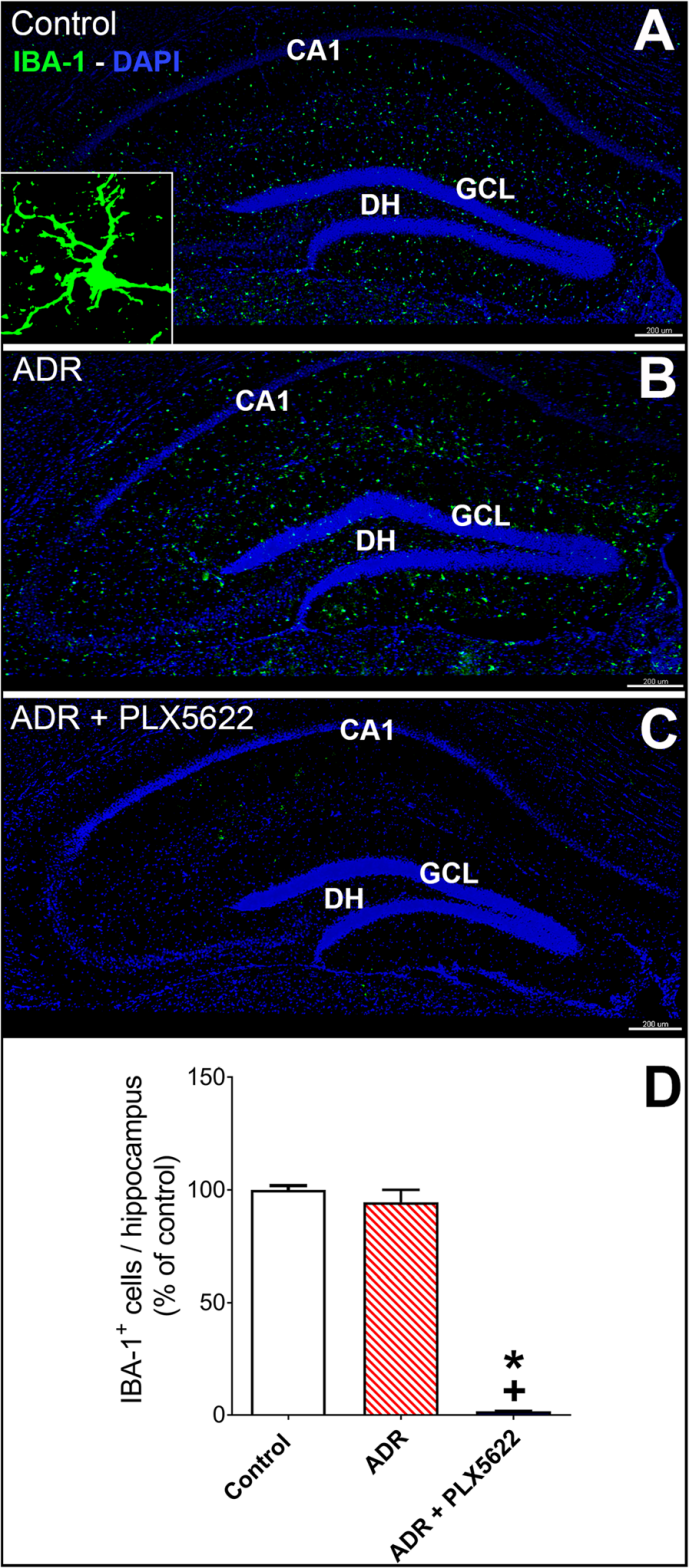
Dietary treatment with CSF1R inhibitor depletes IBA-1^+^ microglia from the ADR-treated hippocampus. Quantification for the IBA-1 immunoreactivity using immunofluorescence staining, laser scanning confocal microscopy and 3D volumetric-based analysis demonstrates that treatment with PLX5622 for 6 weeks eliminates IBA-1^+^ microglia from the ADR-treated (ADR + PLX5622) brains (green, IBA-1; blue, DAPI nuclear counter stain). **a-c** Representative confocal micrographs for IBA-1 immunostaining from the hippocampal dentate gyrus showing dentate hilus (DH), granule cell layer (GCL) and CA1 sub-regions from the control and ADR groups receiving control diet and, ADR-treated mice receiving PLX5622 diet (insert, high resolution image of IBA-1^+^ cell). **d** 3D algorithm-based quantification (Autoquant and Imaris) of IBA-1^+^ microglia show > 96% depletion in the ADR + PLX5622 group. Data are presented as mean ± SEM (*N* = 4–6 mice per group). *P* values are derived from ANOVA and Bonferroni’s post hoc test. *, ^+^ *P* < 0.0001 compared with Control and ADR groups respectively. Scale bars, 200 μm, **a-c** 5 μm, insert

**Fig. 3:**
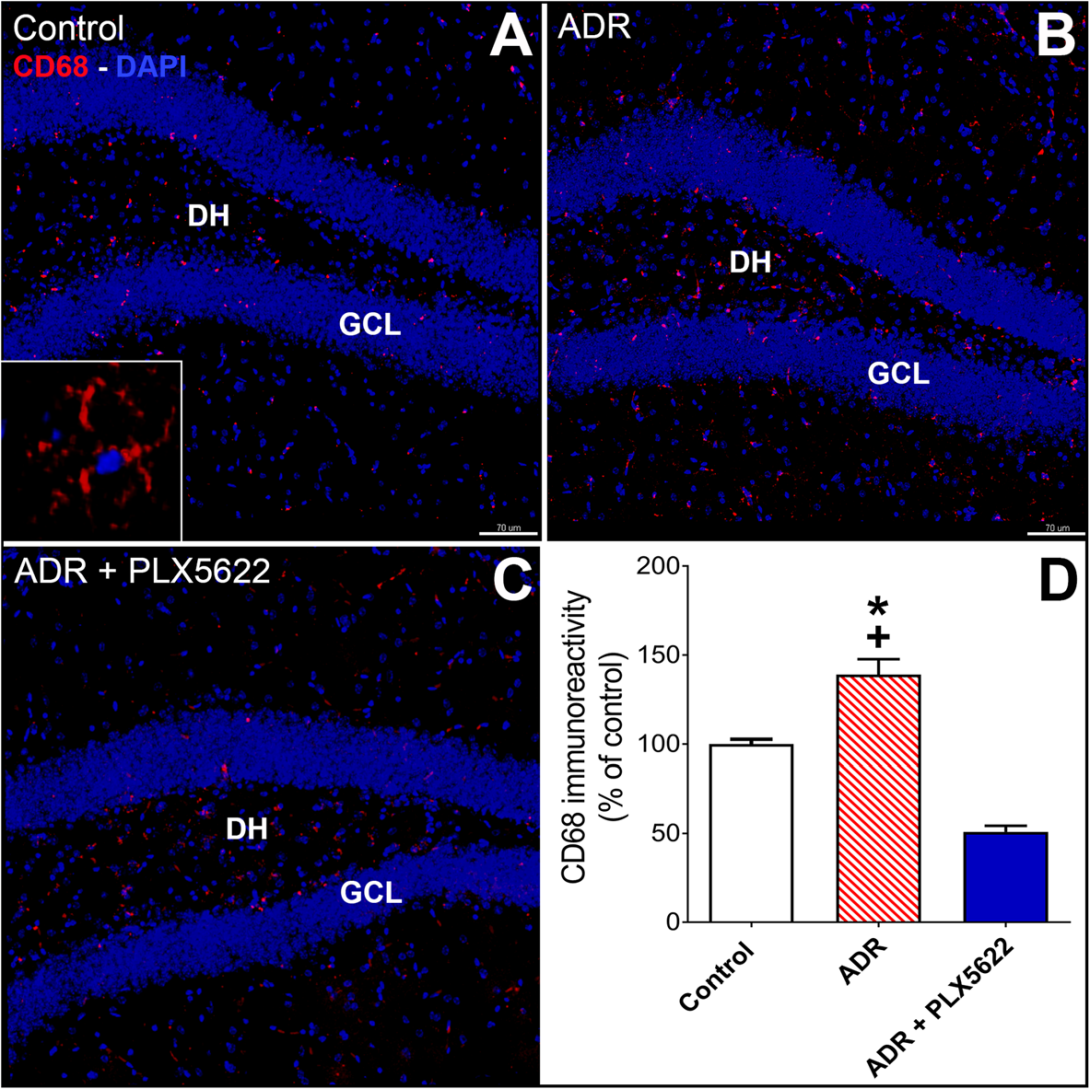
CSF1R inhibitor treatment reduces CD68^+^ activated microglia from the ADR-treated hippocampus. Immunofluorescence staining, confocal microscopy and 3D algorithm-based volumetric quantification show that PLX5622 treatment for 6 weeks significantly reduced CD68^+^ activated microglia (red, CD68; blue, DAPI nuclear counterstain) in the ADR-treated brains. **a-c** Representative confocal micrographs for CD68 immunostaining from the hippocampal dentate gyrus showing dentate hilus (DH) and granule cell layer (GCL) from the Control and ADR groups receiving control diet and ADR-treated mice receiving PLX5622 diet (insert, magnified image of CD68^+^ cell). **d** 3D algorithm-based quantification (Autoquant and Imaris) of CD68^+^ activated microglia show > 60% reduction in the ADR + PLX5622 group. Data are presented as mean ± SEM (*N* = 4–6 mice per group). *P* values were derived from ANOVA and Bonferroni’s post hoc test. *, ^+^ *P* < 0.0001 compared with Control and ADR groups respectively. Scale bars, 70 μm, **a-c** 5 μm, insert

We have demonstrated previously that cancer therapy (cranial irradiation or chemotherapy)-induced microglial activation contributes to cognitive impairments [3–5, 22]. To determine the effectiveness of dietary treatment with CSF1R inhibitor, the number of IBA-1^+^ and CD68^+^ activated microglia was quantified (Figs. 2 and 3). The chronic ADR treatment did not affect the numbers of IBA-1^+^ microglial cells 6 week post-treatment, but ADR-treated mice receiving the PLX5622 diet showed a significant depletion in the number of IBA-1^+^ microglial cells (Fig. 2a-c, F_(2, 15)_ = 266.3, *P* = 0.0001). 3D algorithm-based volumetric quantification of IBA-1^+^ microglial cells showed that CSF1R inhibition led to > 95% reduction in IBA-1 immunoreactivity in ADR-treated brains as compared to Control and ADR groups (Fig. 2d; *P* = 0.0001). Chronic chemotherapy significantly increased the CD68 immunoreactivity of activated microglia that was mitigated by PLX5622 treatment (Fig. 3a-c, CD68, *P* = 0.001 versus Control group). The overall group difference for CD68 immunoreactivity was also significant (F_(2, 15)_ = 61.96; *P* = 0.0001) and PLX5622 treatment significantly ablated CD68 immunoreactivity in the ADR-exposed brain as compared to Control and ADR groups (Fig. 3d, *P* = 0.0001). These data indicate that chemotherapy-induced microglial activation was, at least in part, associated with cognitive impairments.

### CSF1R inhibitor reduced pro-inflammatory signatures in the ADR-treated brain

**Fig. 4:**
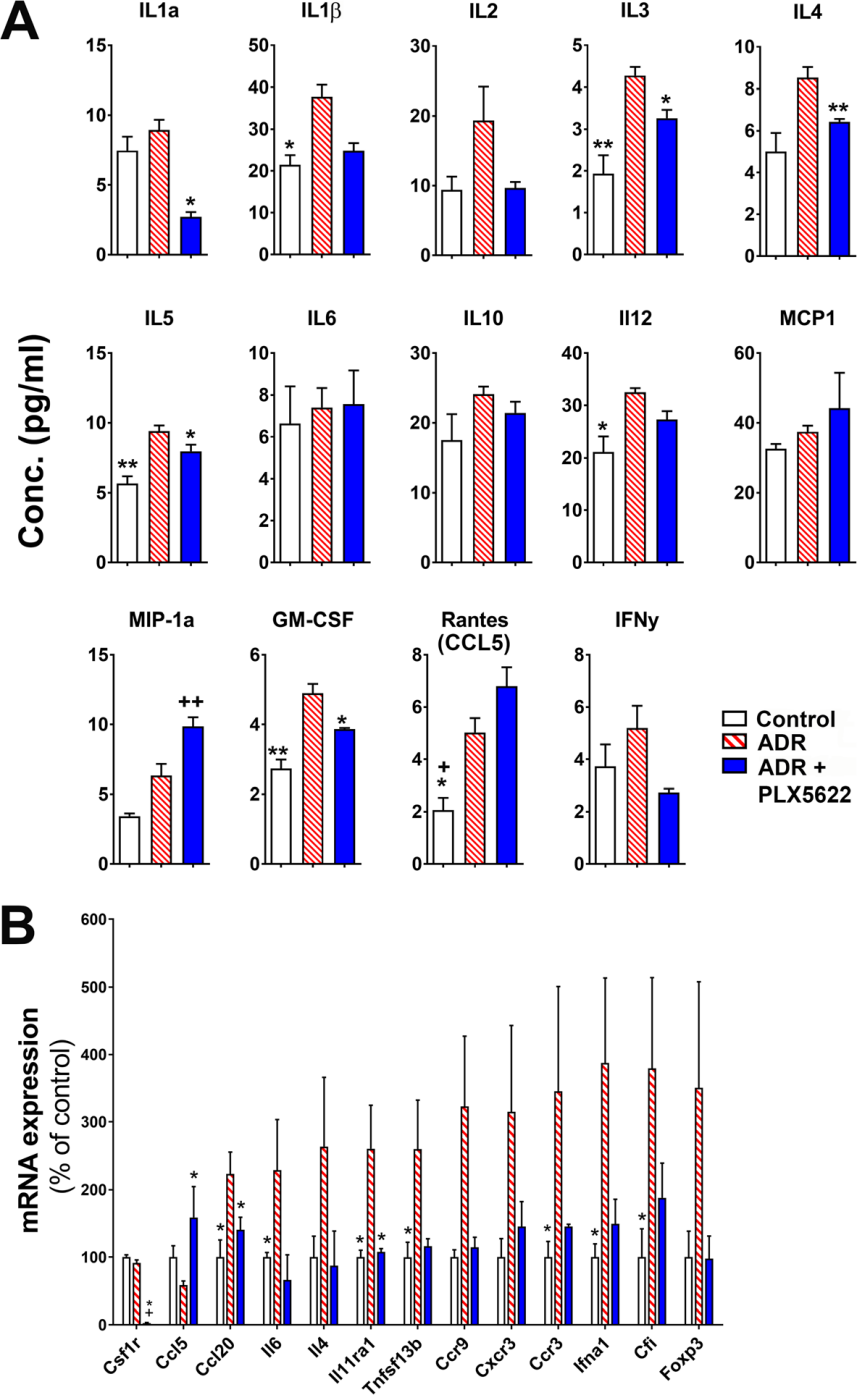
Cytokine profile and gene expression analyses of ADR and ADR-treated mice brains receiving PLX5622. **a** Determination of hippocampal cytokine profile (ELISA) showed significant alterations in the IL-1a, IL-1β, IL-3, IL-4, IL-5, MIP-1a, GM-CSF and Rantes (CCL5) levels (pg per ml of hippocampal suspension) in the ADR and ADR + PLX5622 treated brains compared to Controls. **b** Gene expression analysis of microglial and inflammatory markers from the mice hippocampus show upregulation of pro-inflammatory signatures in the ADR-treated brains. Data are presented as mean ± SEM (*N* = 3 to 5 mice per group). *, *P* < 0.05; **, *P* < 0.01 compared with ADR group. +, *P* < 0.05; ++, *P* < 0.003 compared with control group

The foregoing data indicated that chronic chemotherapy-induced neuroinflammation and cognitive dysfunction could be reversed by CSF1R inhibition. Pro-inflammatory cytokine signaling trigger cascades of events that may lead to persistent microglial activation and disruption of brain function [33]. To corroborate these findings, we carried out multiplex ELISA for cytokines and, gene expression analyses from freshly dissected hippocampal tissues (Fig. 4). Chronic ADR treatment significantly elevated levels of IL-1β, IL-3, IL-5, IL-12, GM-CSF and RANTES (Regulated on Activation, Normal T Cell Expressed and Secreted, CCL5, Fig. 4a). Administration of PLX5622 to the ADR-treated animals led to a significant decline in the levels of IL1α, IL-3, IL-4, IL-5 and GM-CSF while MIP-1a and CCL5 were elevated by PLX5622 treatment. Gene expression analyses showed elevated pro-inflammatory signatures in the ADR-treated brain, including IL-6, IL-4, IL11ra1, Tnfsf13b and Cfi (Fig. 4b). Again, treatment with PLX5622 reduced inflammatory gene expression levels in the ADR-treated brains. Taken together, CSF1R inhibition reduced neuroinflammation in the ADR-treated brains that is linked with improvements in the cognitive function.

### iMG-EV treatment reversed ADR-induced cognitive dysfunction

**Fig. 5:**
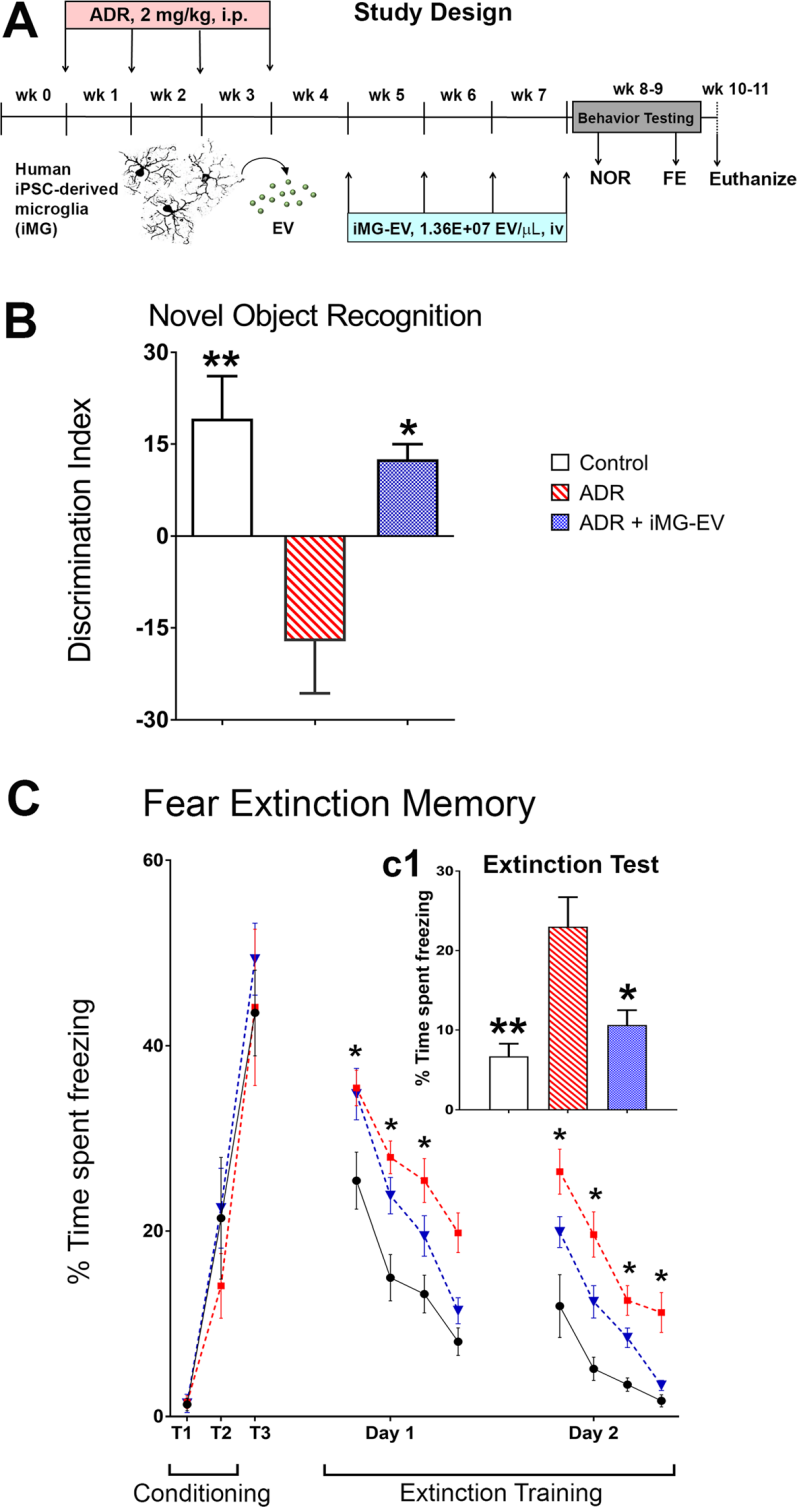
Extracellular vesicles (EV) isolated from human induced pluripotent stem cell (iPSC)-derived microglia (iMG) prevent the development of Adriamycin-induced cognitive dysfunction. **a** Schematic of the experimental design: adult wild type (C57BL/6 J) male mice received chronic Adriamycin (ADR) treatment (2 mg/kg, i.p.) once weekly for 4 weeks. One week after the last ADR injection animal received intravenous (retro orbital vein) injections of iMG-EV (1.36 × 10^7^ EVs per injection in 50 μL volume, once weekly for 4 weeks). One week after iMG-EV injections, mice were administered novel object recognition (NOR) and fear extinction memory (FE) tasks. After completion of cognitive testing, brains were harvested for immunohistochemical analyses. **b** Chronic ADR treatment caused a significantly reduced discrimination index (DI) on the NOR task (**, *P* < 0.002 compared to Controls). ADR-treated mice receiving iMG-EV injections (ADR + iMG-EV) show a significant improvement in performance on the NOR task (*, *P* < 0.01 compared to the ADR group). **c** Neither treatment with ADR nor iMG-EV impaired the acquisition of conditioned fear as indicated by elevated freezing following a series of 3 tone-shock pairings (0.6 mA, T1-T3). 24-h after the conditioning phase, fear extinction training was administered every 24-h (20 tones) for 2 days. All mice showed a gradual decrease in freezing behavior (Day 1–2), however, ADR-treated mice spent a significantly higher percent time in freezing compared to controls (*, *P’s* < 0.01). **c1** 24 h after extinction training, Control and ADR + iMG-EV mice showed abolished fear memory compared to ADR-treated mice receiving vehicle (*, *P* < 0.01; **, *P* < 0.002 compared to ADR group). Data are presented as mean ± SEM (*N* = 8 mice per group). *P* values were derived from ANOVA and Bonferroni’s post hoc test

Our data emphasize the role of microglia in disrupting normal brain function after the exposure to cytotoxic cancer therapy. Our past studies have shown beneficial effects of reducing CNS inflammation by various strategies including CSF1R inhibition [4], reduction of astrogliosis [2] and, stem cell-derived EV treatment [17]. In the next set of experiments, 1 week after last ADR treatment, animals were treated with iMG-derived EV once weekly for 4 weeks via retro orbital vein injection (Fig. 5A; 1.36 × 10^7^ EVs per injection). Control mice receiving EV treatment were not included given it is not clinically relevant and the control brain does not show measurable neuroinflammation pathology. One week after the last EV injection, animals were habituated and administered the NOR spontaneous exploration task. The overall group differences between each treatment cohort were significant (F_(2, 21)_ = 8.914, *P* = 0.002) for the NOR test phase (Fig. 5B). The ADR-treated mice spent significantly less time exploring the novel object compared to Control mice receiving vehicle (*P* = 0.002). iMG-EV treatment significantly improved the performance of ADR treated mice (*P* = 0.01 vs ADR group) as indicated by comparable exploration to the novel objects as in the Control group. Moreover, Wilcoxon matched-pairs signed rank test comparing familiar and novel exploration times revealed significant effect for the Control (*P* = 0.02) and ADR + iMG-EV (*P* = 0.01) groups whereas exploration times for the ADR group were statistically indifferent (Additional file 1: Figure S3).

Our past and current data show that chemotherapy impairs contextual fear conditioning memory (Fig. 1d) [5, 22]. Next, we conducted fear extinction (FE) memory testing to decipher if chemo-treated mice could acquire and subsequently extinguish conditioned fear responses (fear memory consolidation). During the conditioning phase of FE testing, all groups of mice (Control, ADR and ADR + iMG-EV) exhibited comparable associative learning as demonstrated by similar times spent freezing during the tone-shock conditioning phase (Fig. 5C; T_1_-T_3_; 44 to 49% on T_3_). During the subsequent extinction training days, mice were presented with 20 tones per day (5 s intervals) in the same context as the conditioning phase with no foot shock. The ADR-treated animals continued to show increased freezing as compared to the Control and ADR + iMG-EV groups (Fig. 5C; Extinction Training Day 1 and 2, *P* = 0.01). These data indicated that iMG-EV injections to the ADR-exposed animals mitigated impairments in the ability to dissociate the learned response (freezing) to a prior aversive event (tone-shock pairing). Twenty-four hours after completion of extinction training, the mice were administered extinction testing (3 tones, 120 s intervals) in the same testing environment as used for extinction training. The extinction test revealed significant group effects (Fig. 5c1; F_(2, 21)_ = 10.97, *P* = 0.001). ADR mice demonstrated an inability to abolish fear memories during this retrieval testing and again exhibited increased freezing that was ameliorated by iMG-EV injections (Fig. 5 c1; *P* = 0.01 vs ADR group). Moreover, Wilcoxon matched-pairs signed rank test show that Control and ADR + iMG-EV groups spent significantly less time freezing on the test day versus the first training day (Additional file 1: Figure S4, *P* = 0.002, training vs test day). This hippocampus-dependent FE testing paradigm provides a relative invasive measure of elevated anxiety and impairments in fear memory consolidation, and demonstrated that chemotherapy induced impairments similar to a post-traumatic stress disorder that can be abolished by repeated injections with iMG-derived EV.

### iMG-EV treatment attenuates microglial activation

**Fig. 6:**
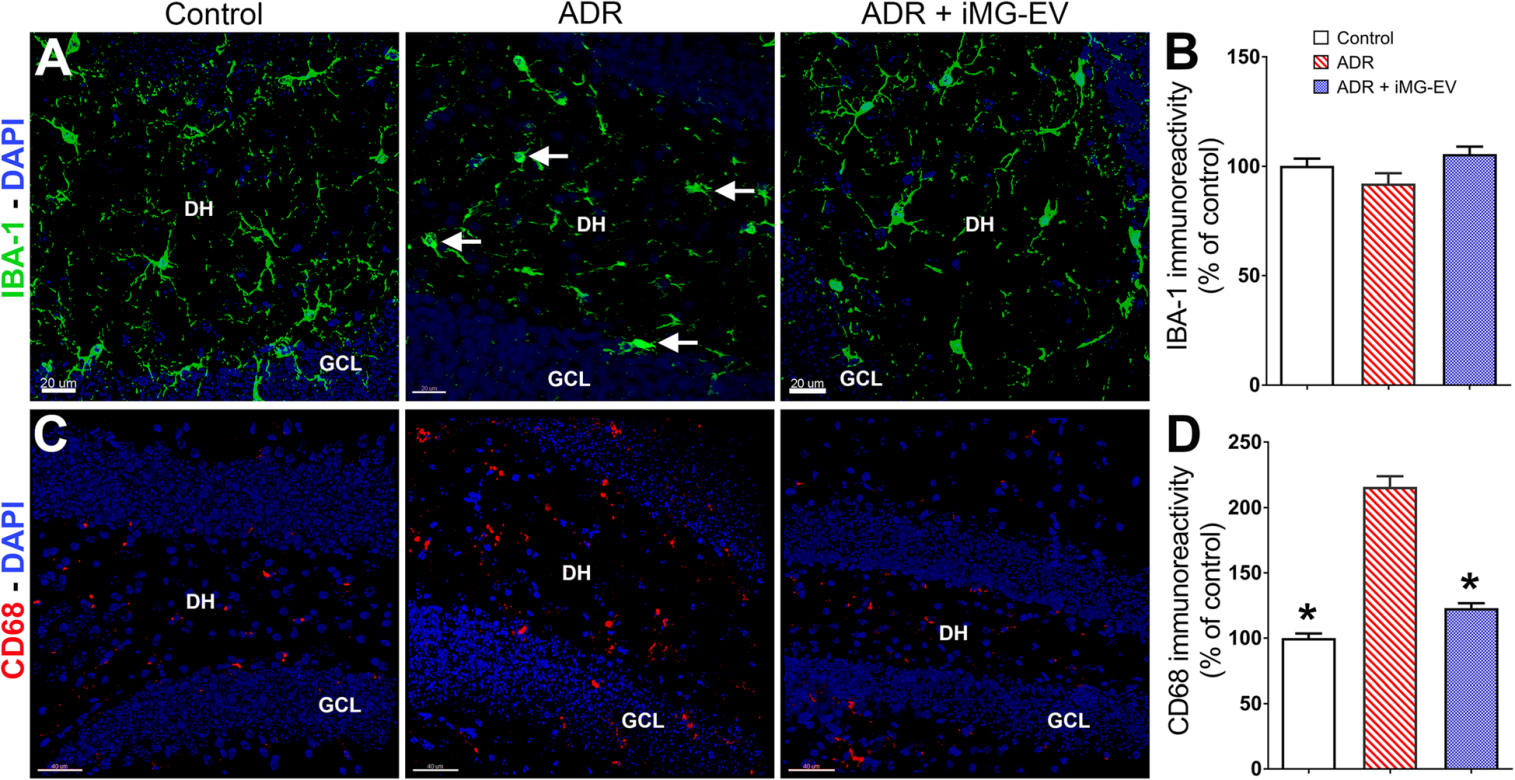
Extracellular vesicles (EV) isolated from human induced pluripotent stem cell (iPSC)-derived microglia (iMG) reduce neuroinflammation. **a**, **b** Immunofluorescence staining, confocal microscopy and 3D algorithm-based quantification for the IBA-1^+^ cells (IBA-1, green; blue, DAPI nuclear counter stain) show that neither chronic ADR treatment, nor retro-orbital vein injections of iMG-EV altered the IBA-1^+^ microglial population in the hippocampus (DH, dentate hilus; GCL, granule cell layer). ADR treated mice (**a**, middle panel) show amoeboid morphology (white arrows) characteristic of microglial activation. **c**, **d** Chronic treatment with ADR elevated CD68^+^ activated microglia in the hippocampus (DH, dentate hilus; GCL, granule cell layer). iMG-EV injections significantly reduced the CD68 immunoreactivity indicating attenuation of inflammation. Data are presented as mean ± SEM (*N* = 6 mice per group). *, *P* < 0.001 compared with ADR group. *P* values were derived from ANOVA and Bonferroni’s post hoc test. Scale bars: **a** 20 μm and **c** 40 μm

iMG-EV treatment-mediated improvements in cognitive function indicate the importance of modulating inflammatory system in the chemo-treated brains. Microglia play a critical role in sustaining learning and memory behaviors [45, 53]. Immunohistochemistry and volumetric quantification were carried out to determine the status of microglia in the ADR treated brain following iMG-EV treatment (Fig. 6). As observed previously (Fig. 2), chronic chemotherapy did not affect the immunoreactivity of IBA-1^+^ microglial cells (Fig. 6a, b). Close evaluation of IBA-1^+^ cells in the ADR-treated brain showed typical amoeboid, round microglial morphology with stout processes (Fig. 6a, white arrows) indicating activation status after ADR treatment. This observation was confirmed by CD68 staining to visualize activated microglia (Fig. 6c). Chronic ADR treatment significantly increased CD68 immunoreactivity compared to controls (*P* = 0.0001). Conversely, iMG-EV treatment significantly reduced activated microglia in the ADR-treated brain (Fig. 6d, *P* = 0.0001). These data demonstrate the neurotoxic role of microglia in the chemo-treated brains and, provide evidence that iMG-EV treatment can attenuate microglial activation and remediate chemobrain.

## Discussion

Cancer survivors experience emotional, behavioral and cognitive decrements long-term post-treatment in the absence of cancer, seriously impacting quality of life [7, 42]. With significant increases in the number of cancer survivors, chemobrain represents a critical survivorship issue with notable absence of clinical recourse. Therefore, strategies to restore cognition and normal brain function following the successful completion of cancer therapies are clearly needed. The current pre-clinical literature and our past data have shown that neurocognitive impairments correlate with elevated pro-inflammatory cytokines and neuroinflammation [5, 22, 40]. The activation of microglia is also linked with several neurodegenerative conditions, including radiation and chemo-therapy-related brain injury. Thus, we targeted common neuropathological sequelae – CNS inflammation – to ameliorate chemotherapy-related cognitive impairments.

Using a relevant rodent model of cranial radiation-induced brain injury, we have shown previously that administration of a highly specific, brain penetrant CSF1R inhibitor (PLX5622) eliminated microglia (> 96%) in the irradiated brain within 3 days post initiation of dietary treatment and, importantly, ameliorated radiation-induced behavioral decrements that were evaluated 4–6 weeks after irradiation [4]. In the current study, as a proof-of-the-concept, we employed a similar strategy using a rodent model of chemobrain to show the effectiveness of CSF1R inhibition. Animals were treated with chronic ADR (doxorubicin) treatment, a commonly used breast cancer chemotherapy, and 72 h later initiated dietary administration of PLX5622 for 4–6 weeks. As previously reported [22], chronic ADR treatment caused significant impairments in the recognition of novelty, shown by inability of animals to recognize new objects or locations on each of the spontaneous exploration, open field tasks. Deficits were also found on the contextual fear-conditioning task, suggesting that neurocognitive deficits were associated with hippocampal-dependent learning and memory function. ADR-treated animals receiving PLX5622 showed significant improvements in performance on all behavioral tasks suggesting cognitive benefits of microglia depletion. We did not include a group of control mice receiving PLX5622 treatment as the data from our laboratory and others have shown that CSF1R inhibition itself did not alter cognitive function [4, 24, 43]. In depth analyses using immunohistochemistry and 3D volumetric-based quantification, cytokine ELISA, and RNA sequencing conclusively determined the impact of microglia depletion in the chemo-treated hippocampus. The chronic ADR treatment did not alter the number of IBA-1 positive microglia, and treatment with PLX5622 eliminated > 95% of microglia in the ADR-treated brains. This is in line with our previous findings showing microglia depletion in the irradiated brain [4]. Similarly, Green and colleagues have shown the effectiveness of CSF1R inhibition in neurodegenerative disease models suggesting that CSF1R inhibition was equally effective in other brain injury scenarios [43, 47]. Quantification of CD68 immunoreactivity, an indicator of activated microglia, in the ADR-treated brain showed about 1.5-fold elevation in inflammation that was reduced significantly by the CSF1R blockade. Treatment with Adriamycin has been shown to elevate plasma TNFɑ that may perturb the blood brain barrier integrity and exacerbate inflammatory cascade in the brain leading to neuroinflammation [48]. Our gene expression data showed elevation in a pro-inflammatory, supra-family member TNF ligand, Tnfsf13b in the ADR-treated brain. Although, we have not tested this directly, ADR-induced infiltrated macrophage/ monocytes in the CNS may also express CD68 in response to the pro-inflammatory environment. Suppression of microglial activation may yield a plethora of effects on neuronal function, as a large body of literature suggests that activated microglia can exert neurotoxic effects via such routes as production of oxidative or nitrosative stress or TNFα and cytokine secretions that may damage neurons and glia [28]. A single injection of ADR has been shown to induce acute elevation (3 to 72 h) in apoptotic response genes, mitochondrial dysfunction and elevated TNFɑ in the brain [48]. Taken together, these studies indicate ADR-mediated early neuropathological alterations that lead to persistent inflammation and disruption in cognitive function at later post-treatment intervals. Microglia have been shown to regulate synaptic integrity by actively remodeling synaptic and perisynaptic environment via complement signaling that could be disrupted when the brain exhibits persistent microglial activation [23]. Our past data have shown the detrimental effects of chronic chemotherapy on neuron structure and spine density that was linked with microglial activation [5]. Although, we did not analyze neuron morphologic parameters in the current study, the ability of CSF1R inhibition to attenuate neuroinflammation provides one plausible explanation for the beneficial neurocognitive outcomes in the ADR-treated brain.

Neurodegenerative or pathological events, including irradiation and chemotherapy, have been shown to induce cytokine-mediated immune response in the CNS [19, 26, 30]. Our multiplex ELISA data showed elevated levels of pro-inflammatory cytokines in the ADR-treated hippocampus including IL-1β, IL-3, IL-5, IL-12 and GM-CSF. CNS is capable of producing low levels of cytokines that modulates the function of neurons, astrocytes and microglia [33]. IL-1β, IL-3, IL-5 and GM-CSF are early indicators of the cranial radiation-induced inflammatory response [30] and studies have reported key roles for these cytokines in promoting neuroinflammation and deterioration of hippocampal-dependent learning and memory formation [29]. IL-1β overexpression has been shown to impair hippocampal-dependent contextual fear memory [29] which is in line with our data showing ADR-induced elevation of IL-1β and impairments in the contextual as well as extinction of fear memories. IL-1β signaling activates microglia and increases pro-inflammatory cytokine responses that could lead to neuroinflammation and cognitive dysfunction [29]. IL-12 is a mediator of inflammatory neurodegenerative conditions including multiple sclerosis [19]. IL-12 is produced by microglia and astrocytes in the brain and triggers the detrimental degenerative consequences in the CNS via STAT4-dependent induction of IFN-y production [19]. Our data show elevation in the IL-12 and IFN-y in the ADR-exposed hippocampus that links microglial activation and cognitive dysfunction. Both IL-1β and IL-12 levels were reduced following PLX5622 treatment, albeit not statistically significant in comparison with the ADR group. GM-CSF is essential for the expansion of pro-inflammatory immune responses. Elevated or dysregulated GM-CSF is also associated with neuroinflammation and brain injury in humans [8]. CSF1R inhibition significantly reduced GM-CSF levels in the brains of chemo-treated animals indicating attenuation of neuroinflammation. Interestingly, the brains of ADR-treated animals receiving PLX5622 show a notable elevation in levels of RANTES expression. RANTES, also known as CCL5, plays neurotrophic and neuroprotective roles in the brain. For example, CCL5 induces proliferation of oligodendrocyte precursors [34], regulates differentiation of astrocytes [14] and, exerts neuroprotective effects against glutamate-, β-amyloid- or HIV protein gp120-induced neurotoxicity [18, 20, 31]. The observed increase in protein and transcript levels of the neuroprotective CCL5 in the brains of PLX5622-treated animals supports our data demonstrating reduced cognitive dysfunction and anti-inflammatory effects of CSF1R inhibition. These findings clearly indicate quenching of chemokine signaling by CSF1R inhibition in the chemo-treated brain that likely dampens the recruitment and/or activation of microglia.

One of the striking findings of this study is the prevention of chemotherapy-induced cognitive impairments by human iPSC microglia-derived EV. Human iPSC-derived microglia (iMG) are highly similar to cultured human adult and fetal microglia in terms of molecular signatures and show functional characteristics of migration, secretion of cytokines and phagocytosis in vitro and in vivo [1, 41]. The basis of this approach stems from our past studies showing the beneficial neurocognitive effects of human neural stem cells (hNSCs) or hNSC-derived EV in reversing cranial radiation-induced brain injury [3, 17]. EV are secreted from cells in nearly all known tissues, and can play a role in maintaining normal homeostasis or in many disease pathologies, including cancer [49]. EV are now recognized as important circulating biomarkers, as well as therapeutic candidates [12]. EV have been shown to display low immunogenicity, can cross the blood-brain barrier, and fuse and deliver cargo to specific cell types in the brain [12]. In our study, repeated intravenous injections (once weekly for 4 weeks) of iMG-derived EV prevented the development of chemotherapy-related cognitive impairments as reflected by increased time-spent exploring the novel object. Moreover, chemotherapy-induced elevation in the microglial activation was attenuated by iMG-EV treatment. Activated microglia and elevated pro-inflammatory cytokines plays disruptive role in fear memory consolidation [45, 53]. Our data show elevated signatures of pro-inflammatory cytokines, including IL-1β and TNF ligand suprafamily member, Tnfsf13b, after chronic ADR exposure that may explain elevated freezing levels (impaired memory consolidation) during the fear extinction trials. ADR-treated mice receiving iMG-EV injections show improvements in fear memory consolidation reflected by reduced time spent freezing during fear extinction training and testing phases. Interestingly, during the cue-phase of fear conditioning (Fig. 1d) we observe comparable elevated freezing in the Control and ADR groups showing intact amygdala function. This freezing behavior was abolished when ADR-treated mice administered extinction training in the same context (Fig. 5C, extinction training day 1 and 2). This data signifies the rigor of our testing platform and demonstrate that hippocampal-amygdala circuit is also disrupted following chemotherapy, and that additional therapeutic strategy of re-switching the chemo-injured CNS microenvironment to less inflammatory and thereby promoting recovery from chemobrain. Indeed, reports suggest beneficial and anti-inflammatory effects of microglia- or immune cell-derived EV on the CNS function. Repeated intra-nasal delivery of macrophage-derived EV loaded with catalase decreased microglial activation in the 6-hydroxydopamine (6-OHDA)-induced acute inflammation model of Parkinson’s disease [27]. An in vitro study showed neuroprotective effects of EV derived from monomeric α-synuclein treated microglia on the MPP-injured cultured neurons [37]. Intracranial delivery of EV isolated from microglia and mesenchymal stem cell co-cultures promoted oligodendrocyte precursor cell differentiation and remyelination whereas inflammatory microglia-derived EV had opposite effects [39]. Our past findings have shown that cranial injections of hNSC-derived EV protected neuronal dendritic structure, spine density and, significantly reduced microglial activation in the irradiated brain [17]. The foregoing reports show equivalent beneficial effects of intra-cranial and systemic delivery of EV. Injected EV have been shown to fuse or co-localize with various CNS subtypes, and deliver bioactive cargo to produce functional effects [10, 17, 39, 52]. Further work is warranted to investigate the molecular cargo (miRNAs, proteins etc.) of iMG-EV to delineate the beneficial anti-inflammatory effects of EV in the chemobrain model.

Taken together, our findings implicate CNS inflammation, particularly microglial activation, as one of the major causal factor in perpetuation of chemobrain. Extensive studies by Green and co-workers have shown no adverse physiological or behavioral effects of short- or long-term depletion of microglia via CSF1R inhibition in acute brain injury, aging and Alzheimer’s disease mouse models [25, 43, 46]. Depletion of microglia prior to neuronal insult aggravated the injury, whereas microglia elimination following the neuronal injury promoted recovery [43]. On the other hand, sustained microglia elimination in the young (1.5 month old) 5xFAD mouse brain prevented plaque formation over extended period of time (7 month of age) whereas re-population of microglia upon CSF1R inhibitor withdrawal lead to robust plaque formation [46]. Similarly, our past study using a clinically relevant irradiation paradigm did not show adverse physiological effects of PLX5622 treatment on the control animals [4]. Moreover, no adverse impact of PLX5622 treatment on neural stem and oligo-progenitor cell proliferation and differentiation was observed [36, 38]. Therefore, we posit that PLX5622 treatment-mediated reduction in microglia and neuroinflammation contributed significantly to restoring cognitive function. The gist of these studies also indicate that depletion of microglia from the injured or neurodegenerative environment with subsequent re-population upon CSF1R inhibitor withdrawal may serve as a useful strategy. A range of CSF1R inhibitors are currently under clinical trials [21] for the treatment of cancers including metastatic breast cancer (NCT01596751), ovarian cancer (NCT01525602), colorectal and pancreatic cancer (NCT02777710), solid tumors (NCT02452424) and, for rheumatoid arthritis (NCT01329991). Thus, CSF1R inhibition strategies may serve dual potentials in killing cancer and protecting the normal tissue function. Whether CSF1R inhibition strategies (short- or long-term) to eliminate microglia or attenuation of CNS inflammatory microenvironment via EV treatment after clinically relevant adjuvant chemotherapy paradigms remain to be determined. Our study showing beneficial neurocognitive and anti-inflammatory effects of attenuating microglial activation support our hypothesis that neuroinflammation is one of the major drivers in chemotherapy-induced cognitive dysfunction. With chemobrain incidence rates as high as 75% in breast cancer survivors, minimally invasive strategies targeting neuroinflammation can provide clinical recourse for this unmet medical need.

## Supplementary information

**Additional file 1.** Supplementary information.

## Data Availability

Correspondence and request for data or materials should be addressed to MMA. PLX5622 drug and iPSC-MG conditioned media should be obtained through a Material Transfer Agreement with Plexxikon, Inc. and Fujifilm Cellular Dynamics, Inc. respectively.
