# Supplementary material for: Attenuation of neuroinflammation reverses Adriamycin-induced cognitive impairments
Source: Acta Neuropathol Commun. 2019 Nov 21;7:186. doi: 10.1186/s40478-019-0838-8 (PMC6868786; doi:10.1186/s40478-019-0838-8)
Supplement: Supplementary file 1 — Additional file 1. Supplementary information. [file 40478_2019_838_MOESM1_ESM.pdf]

Supplementary Information

Allen et al.
Attenuation of neuroinflammation reverses Adriamycin-induced cognitive impairments
DOI : 10.1186/s40478-019-0838-8

Supplement Figures

Suppl. Figure 1

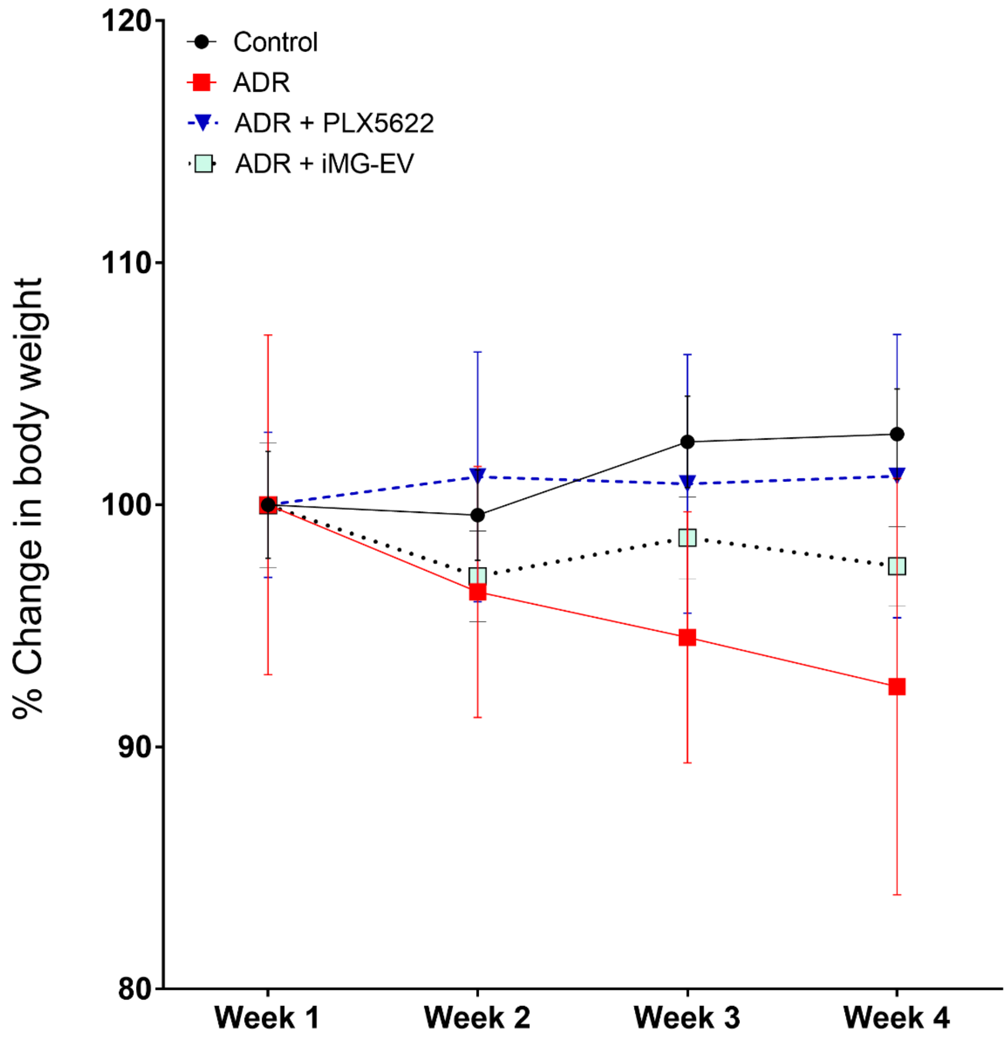

Supplemental Figure S1. Effects of Adriamycin, PLX5622 and iMG-EV treatment on animal body weight. Treatment with Adriamycin, PLX5622 or iMG-EV did not change the body weight of animals significantly. Data are presented as mean ± SEM (*N* = 8 to 10 mice per group) for the percentage change from the initial body weight.

Suppl. Figure 2

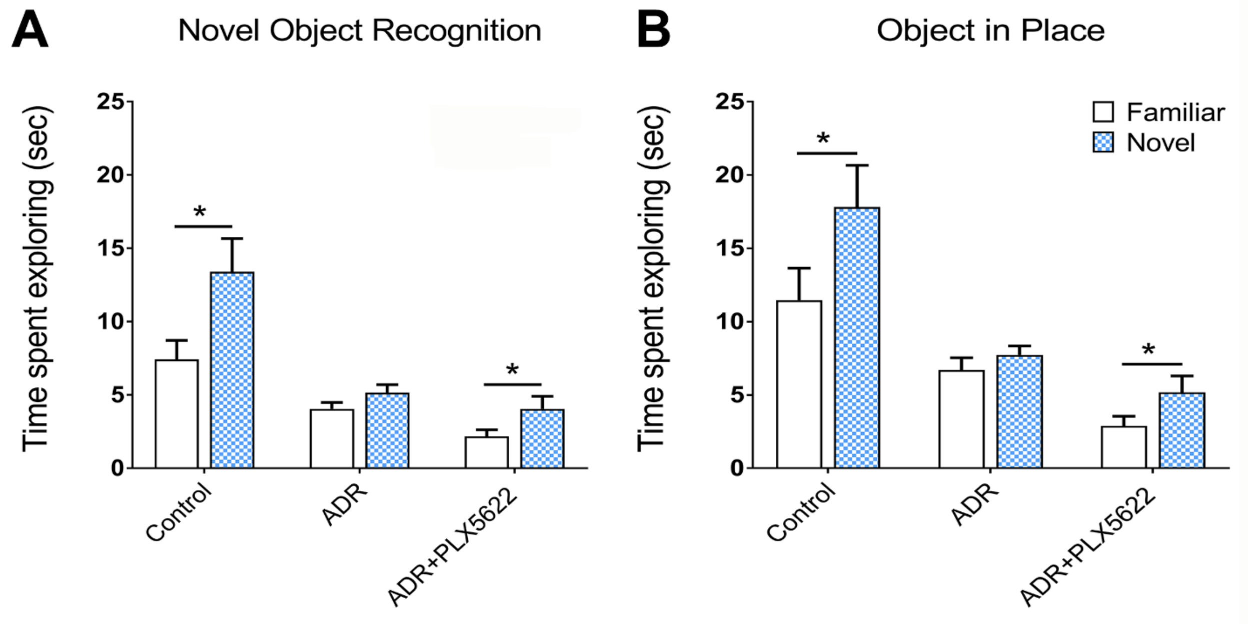

Supplemental Figure 2. Effects of ADR and PLX5622 treatment on total time spent exploring objects during the NOR and OIP tasks. ADR-treated animals spent significantly less time exploring novel objects or places. **A**, Time spent exploring the novel object and, **B**, Time spent exploring novel placements of objects during the test phases of novel object recognition (NOR) and object in place (OIP) tasks, respectively. Control and ADR + PLX5622 treated mice spent significantly more time exploring novel versus familiar object (NOR) or location (OIP) whereas ADR treated mice spent comparable time exploring novel and familiar object or places. Data are presented as mean ± SEM (N = 10 mice per group). P values were derived from Wilcoxon matched-pairs signed rank test. *, $P < 0.002$.

Suppl. Figure 3

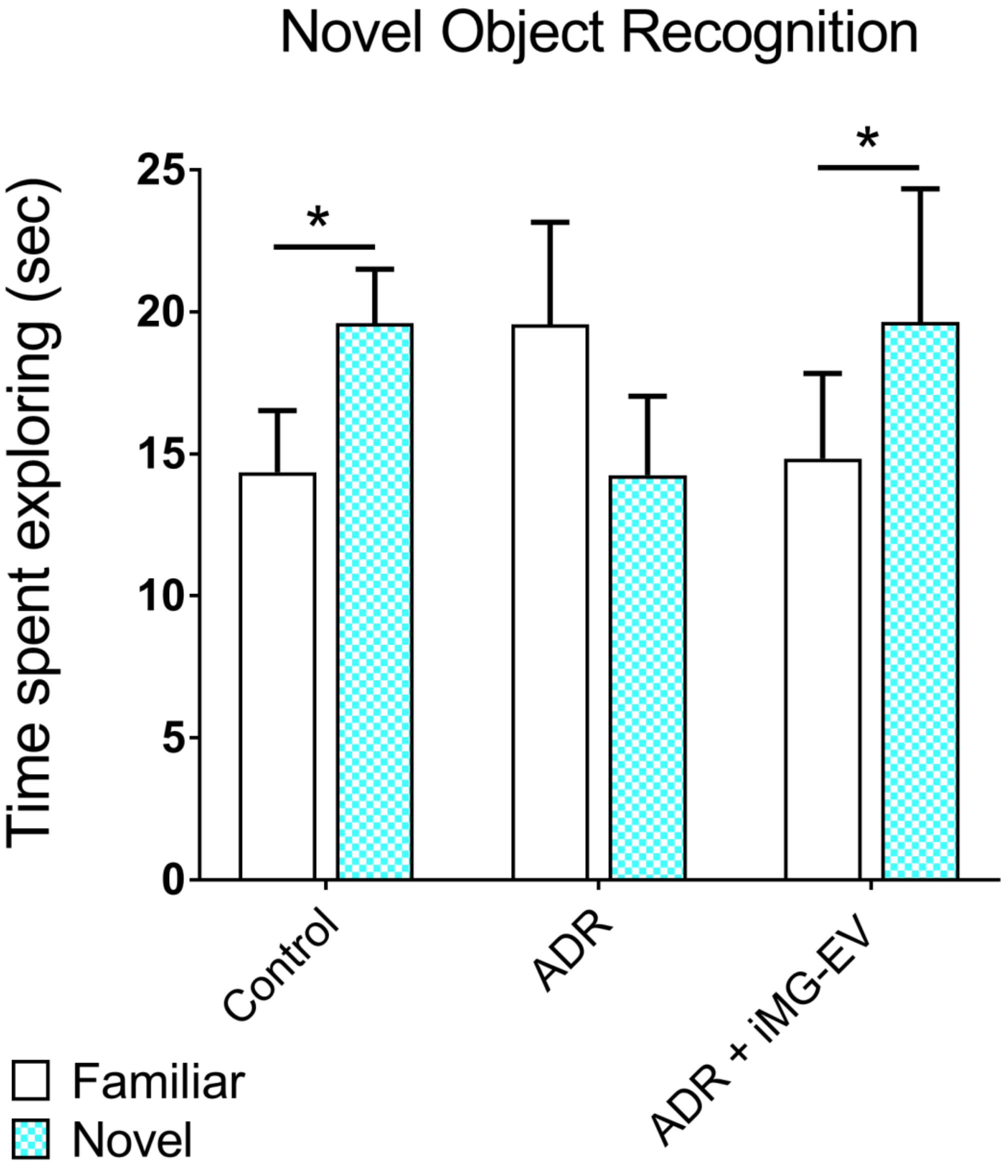

Supplemental Figure 3. Effects of ADR and iMG-EV treatment on total time spent exploring objects during the NOR and OIP tasks. Chronic ADR treatment reduced the time spent exploring novel objects. Time spent exploring the novel object during the test phase of the novel object recognition task shows that Control and ADR + iMG-EV treated mice spent significantly more time exploring novel versus familiar object whereas ADR treated mice spent comparable time exploring novel and familiar objects. Data are presented as mean ± SEM (*N* = 8 mice per group). *P* values were derived from Wilcoxon matched-pairs signed rank test. *, $P < 0.01$.

Suppl. Figure 4

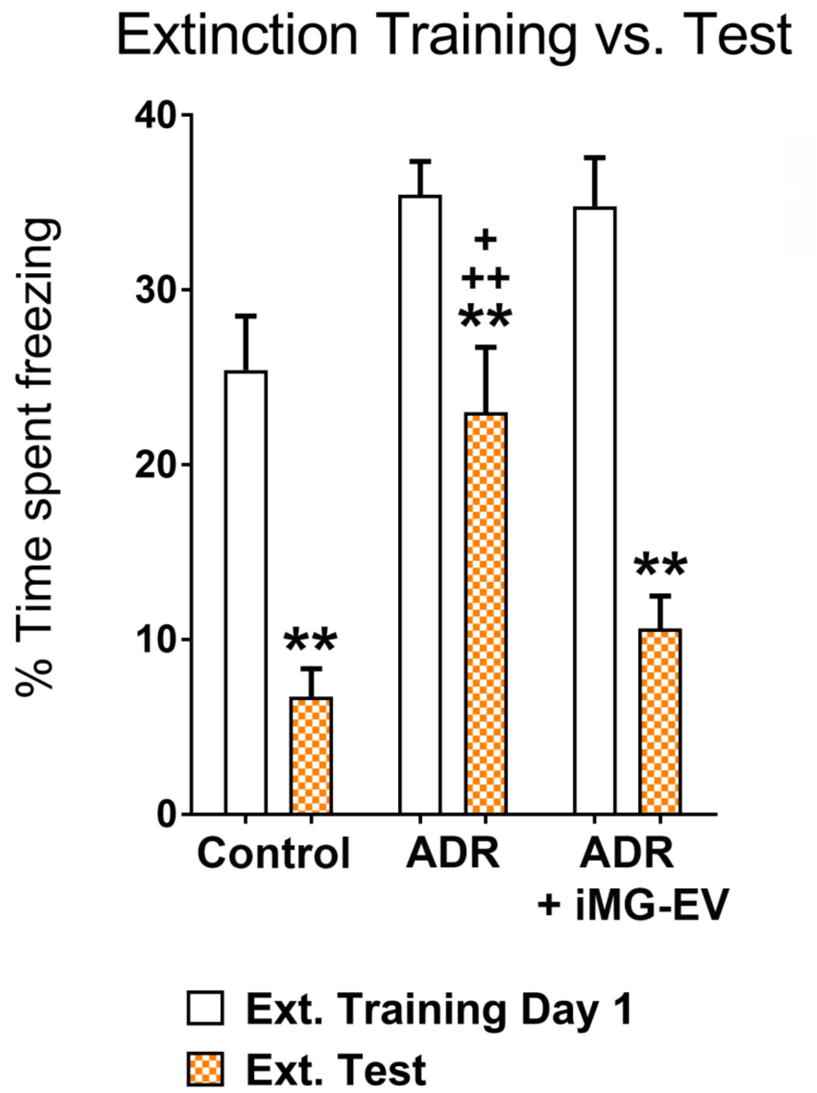

Supplemental Figure 4. Effects of ADR and iMG-EV treatment on percent time spent freezing during the extinction trial and extinction test phases. Disrupted fear memory consolidation following chronic ADR treatment. Control and ADR + iMG-EV treated mice spent significantly less time freezing during extinction test phase versus the first day of extinction training trails. In contrast ADR-treated mice receiving vehicle spent more time freezing during both extinction training and extinction test phases. Data are presented as mean ± SEM (N = 8 mice per group). **, $P < 0.002$ compared to extinction training day 1, Wilcoxon matched-pairs signed rank test. $^{+}$, $P < 0.01$ compared to Control; $^{++}$ $P < 0.002$ compared to ADR + iMG-EV group derived from Bonferroni's post hoc test.

Materials and Methods

Animals and treatments

All animal procedures described in this study are approved by the University of California Institutional Animal Care and Use Committee and, according to the federal (NIH) guidelines. Animals were housed in a pathogen-free facility certified by AAALAC. Six-month old male wild type mice (C57BL/6J, Jackson) were maintained in standard housing conditions (20°C ± 1°C; 70% ± 10% humidity; 12 h:12 h light and dark cycle) and had free access to standard rodent chow and water. Adriamycin (ADR, doxorubicin hydrochloride, Sigma) was dissolved in saline and delivered via intraperitoneal (i.p.) injection once weekly for four weeks (2 mg/kg) as shown in the study design (**Fig. 1A**). For the CSF1R inhibition study, mice were divided into three experimental groups (*N* = 10-12 mice per group): saline treated control mice receiving control chow (Control), ADR-treated mice receiving control chow (ADR) and, ADR-treated mice receiving PLX5622 chow (ADR + PLX5622). 72 hours after the last ADR injection mice were provided the control or PLX5622 chow. CSF1R inhibitor, PLX5622 chow, was provided by Plexxikon (Berkeley, CA) and formulated in AIN-76A standard chow by Research Diets (New Brunswick, NJ) at a dose of 1200 PPM. Control mice received AIN-76A chow without PLX5622. All mice were maintained on their respective PLX5622 or control diet throughout the duration of the study.

Extracellular vesicles (EV) were isolated from human iPSC-derived microglia. Human microglia (iMG) were differentiated by a simplified method from human iPSC-derived mesoderdermal, hematopoietic stem cells as described (1,2). RNA sequencing, phagocytosis, and transplantation studies validated the functional microglial characteristics of these cells. Briefly, large batches of conditioned medium were collected by Cellular Dynamics, Inc. Research and Development Laboratory (Madison, WI) during the maturation phase (days 28 to 35) of the differentiated iMG culture and refrigerated conditioned media was shipped to UCI for the isolation of EV. EV isolation was carried out using the ultracentrifugation protocol as described in detail (Thery). Briefly, large cell debris was removed by sequential centrifugations using increasing speeds (300 × g, 5 min; 2,000 × g, 10 min; 10,000 × g, 30 min; all at 4 °C). At each step the pellet was discarded and the supernatant carried forward. After filtration from 0.2 µm filter, EV were pelleted by ultracentrifugation using 100,000 × g (70 min, 4 °C). The pellet was then washed in sterile PBS to remove contaminating proteins and re-pelleted by centrifugation at 100,000 × g for an additional 70 min. EV quantity and size were determined using a Zetaview particle analyzer (ZetaView PMX 110; Meerbusch, Germany). The iMG conditioned media yielded a total of 7.07×10^{11} EV per ml with the mean diameter of 65 nm. The purified EV were stored in sterile phosphate buffered saline (PBS, 100 mM, pH 7.4, Gibco) at 4°C. Animals were divided into three groups (*N*

= 8 mice per group): Controls receiving PBS (Controls), ADR-treated receiving PBS (ADR) and ADR receiving iMG-EV injection via retro-orbital sinus route of administration once weekly for four weeks (1.36×10^7 EV per 50 µL per injection; ADR + iMG-EV). We did not observe significant effects of any of these treatments on animal body weights (**Suppl. Fig. 1**).

Cognitive testing

To determine the effect of CSF1R inhibition on cognitive function after chronic chemotherapy, mice were administered behavioral testing four weeks after the initiation of PLX5622 treatment. Testing spanned over three weeks including the spontaneous exploration tasks Novel Object Recognition (NOR) and Object in Place (OiP), followed by the contextual and cued fear conditioning (FC) task. The NOR task evaluates episodic recognition memory through measuring the preference of mice to investigate novel environmental changes, whereas the OiP task evaluates associative recognition memory (3,4). Both tasks were conducted as described previously (5). Briefly, NOR and OiP testing occurred in a dimly lit (48 lux) test arena (30 × 30 × 30 cm) containing fresh corn cob bedding and was video recorded from the ceiling. All bedding was replaced and the arena was thoroughly cleaned with 10% ethanol between trials. For the NOR task mice were initially habituated to the empty arena for three days (10 min/day). The following testing day, two plastic objects (differing in color, shape and size) were magnetically affixed 16 cm apart in the arena and the mouse was allowed five minutes to explore the objects. The mouse was returned to the home cage for five minutes while one familiar object was substituted for a novel object (both objects were cleansed with 10% ethanol). The mouse was then returned to the arena for five minutes of further exploration. Three days after completion of the NOR task, OiP task was administered, including, two days (10 min/day) of habituation. On the third day, mice explored an arena with four unique objects for five minutes before briefly returning to their home cage (5 min). All objects were cleansed with 10% ethanol and the location of two objects was swapped before the mouse was returned for five more minutes of exploration. Videos of both tasks were scored to obtain times spent interacting (nose within 2 cm) with familiar versus novel (or relocated) objects by observers blind to the experimental groups to avoid bias. The discrimination index was then calculated for each mouse from these values: [(Novel/Total exploration time) – (Familiar/Total exploration time)] × 100. After completion of spontaneous exploration tasks, the FC task was administered in three sequential phases over three days including a training phase (5 tone and shock pairings, a context test for 5 min and a cue for 5 min test as described previously (6,7).

For the iMG-EV treatment study, mice were administered behavior testing one week after the last EV injection (5 weeks after the last ADR treatment). These tests include NOR and fear extinction (FE) memory testing. NOR testing was carried out as described above. To determine if chronic chemotherapy or EV treatment affects amygdala-hippocampal circuit-dependent fear conditioning learning and fear memory consolidation, we performed a series of FE assays modified to be reliant on hippocampal function (8,9) . Testing occurred in a behavioral conditioning chamber (17.5 × 17.5 × 18 cm, Coulbourn Instruments) with a steel slat floors (3.2 mm diameter slats, 8 mm spacing). Throughout the conditioning, memory consolidation (extinction training) and extinction testing phases, the bottom acrylic collection plate was scented with a spray of 10% acetic acid in water. For the initial fear conditioning phase (day 1), mice were allowed to habituate to the chamber for two minutes. Three pairings of an auditory conditioned stimulus (16 kHz tone, 80 dB, lasting 120 sec; CS) co-terminating with a foot shock unconditioned stimulus (0.6 mA, 1 sec; US) were presented at two minute intervals. On the following two days of extinction training, mice were initially habituated to the same context for two minutes before being presented with 20 non-US reinforced CS tones (16 kHz, 80 dB, lasting 120 sec, at 5 sec intervals). On a final day of fear testing mice were presented with only three non-US reinforced CS tones (16 kHz, 80 dB, lasting 120 sec) at two minute intervals in the same context. Freezing behavior was recorded with a camera mounted above the chamber and scored by an automated measurement program (FreezeFrame, Coulbourn Instruments). FreezeFrame algorithms calculate a motion index for each frame of the video, with higher values representing greater motion. An investigator blinded to the experimental groups set the motion index threshold representing immobility for each animal individually, based on identifying a trough separating low values during immobility and higher values associated with motion. Motion index thresholds were set between 14-15 for all animals. Freezing behavior was defined as continuous bouts of one second or more of immobility. The percentage of time each mouse spent freezing was then calculated for each phase of the fear response testing.

Immunohistochemistry, confocal microscopy and volumetric quantification

After completion of behavioral testing, mice were deeply anesthetized using isoflurane and euthanized via intercardiac perfusion using saline with heparin (10 U/ml, Sigma) followed by 4% paraformaldehyde PBS (ACROS Organics, NJ). Brains were cryoprotected using a sucrose gradient (10-30%) and sectioned coronally into 30 μm thick sections using a cryostat (Leica Microsystems, Germany). For each endpoint 3-4 representative coronal brain sections from each of 4-6 animals per experimental group were selected at approximately 15 section intervals to

encompass the rostro-caudal axis from the middle of hippocampus (2.1 to 2.9 mm from bregma) and stored in PBS. Free floating sections were first rinsed in PBS, blocked for 30 min in 4% (w/v) bovine serum albumin (BSA) and 0.1% Triton X-100 (TTX). For the immunofluorescence analysis of microglia, the following primary and secondary antibodies and dilutions were used: rabbit anti-IBA-1 (1:500, Wako), rat anti-mouse CD68 (1:500, AbD Serotec), donkey anti-rabbit or anti-mouse conjugated with Alexa Fluor 488 or 594 (Life Technologies/Invitrogen). Tissues were DAPI nuclear counterstained (15 µm in PBS) and mounted with gold slow fade/antifade mounting medium (Life Technologies). IBA-1$^+$ cells were visualized under fluorescence as green, CD68$^+$ cells as red and, nucleus as blue.

Immunofluorescent sections were imaged using Nikon Eclipse Ti C2 microscope to obtain 20 to 30 z stacks (1024 × 1024 pixels, 0.5 to 1 µm each) using 20 and 40× PlanApo oil-immersion lens (Nikon). For quantification of IBA-1$^+$ and CD68$^+$ cells, 3D deconvolution and reconstruction was carried out using the AutoQuantX3 algorithm (MediaCybernetics). Deconvolution combined with 3D reconstruction yields higher spatial resolution images for the immunofluorescent cell bodies and stellae ((10,11)). Quantification was facilitated using Imaris filament and spot tool (v9.2, Bit Plane Inc., Switzerland) that detect immunostained puncta within 3D deconvoluted image stacks based on a predefined diameter and red/green channel intensity threshold. IBA-1 and CD68 data are expressed as mean immunoreactivity (percentage) relative to the vehicle-treated controls.

Cytokine and gene expression analyses

Freshly dissected hippocampi from each brain (N = 3-5 per group) were washed and homogenized with 200 µl of PBS containing complete protease inhibitor cocktail (Sigma) using a motorized pellet pestle (Sigma) at 4°C. Supernatants were collected after serial washing in PBS (500 × g and 15,000 × g, 5 minutes each at 4°C) and shipped to Quansys Biosciences (Logan, UT) for the multiplex analysis of cytokines using Q-Plex 14 cytokine array kit. Positive readouts were reported and plotted as the mean ± SEM. For the gene expression analysis, total mRNA was extracted from the freshly dissected hippocampi using the RNA Plus Universal Mini Kit (Qiagen), and were hybridized and multiplexed with NanoString probes according to the manufacturer's instructions. Microglial function and pro-inflammatory genes were analyzed using the NanoString nCounterTM mouse immunology panel (NanoString Technologies). Counts for target genes were normalized to housekeeping genes (EEF1G, G6PDX, HPRT, POLR1B, POLR2A, PPIA, RPL19, SDHA and TBP) to account for variability in RNA content. Background

signal was calculated as a mean value of the negative hybridization control probes. Gene expression values were presented as percentage of vehicle-treated control group.

References

1. Abud EM, Ramirez RN, Martinez ES, Healy LM, Nguyen CHH, Newman SA*, et al.* iPSC-Derived Human Microglia-like Cells to Study Neurological Diseases. Neuron **2017**;94(2):278-93 e9 doi 10.1016/j.neuron.2017.03.042.
2. McQuade A, Coburn M, Tu CH, Hasselmann J, Davtyan H, Blurton-Jones M. Development and validation of a simplified method to generate human microglia from pluripotent stem cells. Mol Neurodegener **2018**;13(1):67 doi 10.1186/s13024-018-0297-x.
3. Barker GR, Bird F, Alexander V, Warburton EC. Recognition memory for objects, place, and temporal order: a disconnection analysis of the role of the medial prefrontal cortex and perirhinal cortex. J Neurosci **2007**;27(11):2948-57 doi 10.1523/JNEUROSCI.5289-06.2007.
4. Barker GR, Warburton EC. When is the hippocampus involved in recognition memory? J Neurosci **2011**;31(29):10721-31 doi 10.1523/JNEUROSCI.6413-10.2011.
5. Parihar VK, Allen BD, Tran KK, Chmielewski NN, Craver BM, Martirosian V*, et al.* Targeted overexpression of mitochondrial catalase prevents radiation-induced cognitive dysfunction. Antioxidants & redox signaling **2015**;22(1):78-91 doi 10.1089/ars.2014.5929.
6. Acharya MM, Martirosian V, Chmielewski NN, Hanna N, Tran KK, Liao AC*, et al.* Stem cell transplantation reverses chemotherapy-induced cognitive dysfunction. Cancer Res **2015**;75(4):676-86 doi 10.1158/0008-5472.CAN-14-2237.
7. Christie LA, Acharya MM, Parihar VK, Nguyen A, Martirosian V, Limoli CL. Impaired cognitive function and hippocampal neurogenesis following cancer chemotherapy. Clinical cancer research : an official journal of the American Association for Cancer Research **2012**;18(7):1954-65 doi 10.1158/1078-0432.CCR-11-2000.
8. Milad MR, Quirk GJ. Fear extinction as a model for translational neuroscience: ten years of progress. Annual review of psychology **2012**;63:129-51 doi 10.1146/annurev.psych.121208.131631.
9. Schubert I, Ahlbrand R, Winter A, Vollmer L, Lewkowich I, Sah R. Enhanced fear and altered neuronal activation in forebrain limbic regions of CX3CR1-deficient mice. Brain Behav Immun **2018**;68:34-43 doi 10.1016/j.bbi.2017.09.013.
10. Acharya MM, Green KN, Allen BD, Najafi AR, Syage A, Minasyan H*, et al.* Elimination of microglia improves cognitive function following cranial irradiation. Scientific reports **2016**;6:31545 doi 10.1038/srep31545.
11. Acharya MM, Baulch JE, Lusardi TA, Allen BD, Chmielewski NN, Baddour AA*, et al.* Adenosine Kinase Inhibition Protects against Cranial Radiation-Induced Cognitive Dysfunction. Frontiers in molecular neuroscience **2016**;9:42 doi 10.3389/fnmol.2016.00042.
